# Securing rights and nutritional health for persons with intellectual disabilities – a pressing challenge

**DOI:** 10.29219/fnr.v62.1268

**Published:** 2018-06-07

**Authors:** Svein Olav Kolset, Marianne Nordstrøm, Sigrun Hope, Kjetil Retterstøl, Per Ole Iversen

**Affiliations:** 1 Department of Nutrition, Institute of Basic Medical Sciences, University of Oslo, Oslo, Norway; 2 Frambu Resource Centre for Rare Diagnosis, Siggerud, Norway; 3 Unit for Inborn and Hereditary Neuromuscular Disorders, Department of Neurology, Oslo University Hospital, Oslo, Norway; 4 Department of Neuro Habilitation, Oslo University Hospital, Oslo, Norway; 5 Lipid Clinic, Oslo University Hospital, Oslo, Norway; 6 Department of Haematology, Oslo University Hospital, Oslo, Norway; 7 Division of Human Nutrition, Stellenbosch University, Tygerberg, South Africa

**Keywords:** Intellectual disabilities, Nutrition, Health, Obesity, Staff nutritional competence, Specific syndromes, Nutritional policies

## Abstract

Persons with intellectual disabilities (ID) are dependent on nutritional policies that have so far not been addressed in a systematic and health-promoting manner in Norway and other nations with a high socioeconomic standard. In many poor countries, such issues have not even been raised nor addressed. Nutritional issues facing persons with ID include the risk of both underweight and overweight. Deficiency in energy, vitamins, essential fatty acids and micronutrients can increase the risk of additional health burdens in already highly vulnerable individuals. According to the World Health Organization, the obesity rates have tripled worldwide the last decades, and recent studies suggest that the prevalence of obesity is even higher for persons with ID than in the general population. This implies additional burdens of life style diseases such as diabetes and hypertension for adults with ID. According to the Diagnostic and Statistical Manual of Mental Disorders (DSM)-5, this group is characterized by intellectual difficulties as well as difficulties in conceptual, social, and practical areas of living. Their reduced intellectual capacity implies that they often have difficulties in making good dietary choices. As a group, they are dependent upon help and guidance to promote a healthy life style. To improve their health, there is a need for improved national services and for more research on lifestyle and nutritional issues in persons with ID. From a human rights perspective, these issues must be put on the agenda both in relevant UN fora and in the respective nations’ health policies.

Approximately 1% of the world population have intellectual disabilities (ID) (1). Life expectancies for this group have been increasing (2), but they still have a lifelong, severely increased mortality rate and reduced life time expectancy (3). According to the World Health Organization, the world wide obesity rates have tripled since 1975 (4, 5, 6), and in a recent large study, the prevalence of obesity is three times higher in persons with ID than in the general population (6). In line with this, type 2 diabetes was more than twice as prevalent in persons with ID as in the general population (6, 7). In a national Swedish study describing health concerns in persons with ID over the age of 55 years, higher incidence of several nutrition-related health problems was reported, for example, iron deficiency twice as prevalent, fluid and electrolyte disturbances twice as prevalent and functional digestive problems were five times more prevalent (7). This study also confirmed the increased risk of type 2 diabetes. Persons with ID belong to all age groups, and nutritional challenges have been found in children, adolescents and elderly persons (7, 8). With higher life expectancy, the elderly might increasingly belong to the group of ID, and their needs for nutritional support warrant attention. A recent study reported that dehydration and malnutrition were the second most common causes of death in elderly persons with ID (9), and another study reported a three times higher risk of dying from nutritional, metabolic, endocrine and digestive diseases than the general population (10). To reduce mortality and comorbid diseases, it is urgent to target the causes of the nutrition-related health challenges.

Nutritional issues facing persons with ID include the risk of both underweight and overweight. The former is often the case with persons in the lower IQ categories (11, 12) and in diagnoses associated with dysphagia like cerebral palsy. Overweight and obesity are most frequently found in persons with mild to moderate ID, in females and in some genetic syndromes. From a Norwegian study, high prevalence of abdominal obesity was found in adult persons with Prader–Willi syndrome, Williams syndrome and Down syndrome (13). Health consequences of obesity will differ between different syndromes with ID, but the need for preventive measures to avoid overweight is an obvious general task for those in charge of health care for persons with ID. Possible causes underlying the nutritional challenges include low intakes of fruit and vegetables (14), high intakes of soft drinks (15), and eating habits with high frequency of snacking (16). Selective eating pattern is common in persons with autistic traits (17). Other contributing factors include low levels of physical activity and exercise; limited availability of community leisure facilities (18); greater use of psychotropic medication; and lack of knowledge, social support and guidance in healthy life style from caregivers. In addition, many persons with ID have a predisposition for digestive problems, with swallowing difficulties, and weight problems.

ID is, according to DSM-5, characterized by intellectual difficulties as well as difficulties in conceptual, social, and practical areas of living. Their reduced intellectual capacity implies that they often have difficulties in making good dietary choices. As a group, they are dependent upon help and guidance to promote a healthy life style with a balanced dietary intake, increasing energy expenditure and sufficient dietary advice.

It is still largely unknown how many persons with ID are registered in different countries and how many of them have been diagnosed with genetic diseases and syndromes. More research is warranted. We need to adopt preventive nutritional measures addressing the specific needs of the group, in order to improve outcome measures (19). More data are needed regarding different health conditions for persons with ID.

## Human rights, ID, and food

The group’s nutritional challenges need to be addressed by health professionals, researchers, community workers, family and other concerned parties, as the right to adequate food is a fundamental human right. The Universal Declaration of Human Rights from 1948, article 25, states: ‘Everyone has the right to a standard of living adequate for the health and well-being of himself and of his family, *including food*, clothing, housing and medical care and *necessary social services*’. It is therefore important to address the challenges of securing persons with ID such legal rights.

The U.N. Convention on the Rights of Persons with Disabilities (https://www.un.org/development/desa/disabilities/convention-on-the-rights-of-persons-with-disabilities.html) from 2006 addressed a wide range of issues with the purpose ‘to promote and ensure the full and equal enjoyment of all human rights and fundamental freedoms by all persons with disabilities, and to promote respect for their inherent dignity’ (From Article 1: Purpose). In this article, it is also stated that: ‘Persons with disabilities include those who have long-term physical, mental, intellectual or sensory impairments which in interaction with various barriers may hinder their full and effective participation in society on an equal basis with others’. In this UN document, emphasis is on physical disabilities and the challenges related to legal rights, transportation, building access, use of sign language for blind and deafblind, independent living, access to information and other relevant issues for persons with disabilities. Importantly, the specialized needs of persons with ID are not specifically addressed, reflecting their generally marginalized status.

Article 25 on health does, however, indirectly address many issues that are of high relevance for improving nutritional measures for persons with ID. Of particular relevance is the statement: ‘Provide health services needed by persons with disabilities and specifically because of their disabilities, including early identification and intervention as appropriate, and services designed to minimize and prevent further disabilities’. As a consequence thereof, persons with ID and nutritional challenges have the right to be early identified and to services that prevent further disabilities. Both obesity and lack of essential nutrients will represent additional burdens to their disabilities.

Health issues related to disabilities are receiving increasing attention. Recently *The Lancet* addressed The Day of persons with disabilities (20) with an editorial entitled ‘Securing the right to health for children with disabilities’. The background in the editorial is struggles experienced by children with disabilities as a result of a society that marginalizes and excludes persons with ID. The editorial makes note of UNICEF recommendations with two main approaches: firstly, mainstreaming disability within general initiatives, and secondly, addressing specific needs through targeted interventions.

The UN document ‘The 2030 Agenda for sustainable development’ (https://sustainabledevelopment.un.org/post2015/transformingourworld/publication) is focused on hunger, food security, development, education and several other issues. Persons with disabilities are included in several goals including social and economic development, education and health. Of importance is also the issue of data, monitoring and accountability, where the generation of reliable data and statistics also for persons with disabilities is specifically mentioned.

Further support for the right to food and the right to health has also recently been provided by the American Association for the Advancement of Science and Human Right Coalition (21). In this document, it is stated that ‘access to nutritious food must not be viewed as a luxury for the wealthy, but instead a fundamental right for all’. It is further stated that ‘people with disabilities have the right to health, safety and education’. Of interest is also the issue of racial minorities and right to health where the document states that ‘the underrepresentation of minorities in clinical studies affects their well-being’. Similar underrepresentation in research is also highly relevant for persons with ID.

Thus, it is clear that both human rights and other international policy documents support that persons with ID have the right to healthy nutrition and that medical and social care need to take into account their special needs.

## National services, ID, and nutrition

The national services provided to persons with ID diverse between countries to a very large extent. Economic and social development, level of income, the inclusion or lack of such persons with ID in health and social programs differ among countries. In many countries, young and adults with ID are invisible, due to social and cultural attitudes of discrimination and shame linked to having a family member with ID. About 80% of the 1 billion people globally with different types of disabilities live in poor countries. In a recent paper (22), it is pointed out that disability has not received the same type of interest as specific diseases like HIV/AIDS, tuberculosis and malaria. The authors discuss several of the health problems facing persons with disabilities, in particular in developing countries, and point to the challenges for health services of ‘reducing barriers such as physical access, negative attitudes, low awareness and increasing the competence of healthcare workers to treat persons with disabilities in an equitable way’.

There are very few studies from low-income countries regarding the nutritional status of adults with ID, and it is uncertain to which degree data from high-income countries may be extrapolated to low-income counties. Still, many low- and medium-income countries have an increasing obesity problems (4). Furthermore, a study of children with ID was equally affected by the obesity epidemic as the general population in low-income countries and at increased risk of underweight (23). Also, we find it interesting that participants in special Olympics, which probably are highly physically fit compared with other persons with ID, as many as 28% from low-income economies and 42% from high-income economies, had BMI levels outside of the normal range. Furthermore, the low-income countries had higher rates of underweight and the high-income countries had higher rates of obesity (24) and are likely related to differences in the services and the health care systems in the different countries.

Persons with ID face challenges in their daily lives that need to be addressed by health professionals, researchers, community workers and other concerned parties. Nutritional policies have so far not been addressed in a systematic and health-promoting manner in Norway and other nations with a high socioeconomic standard. In many poor countries, such issues have not even been raised nor addressed. Firstly, our experience from Norway is that there is a lack of data on nutritional status for young and adults with ID, practical measures and manuals for preventive nutritional work in the municipalities and research on nutrition and health for persons with ID (13, 25). To promote a healthy diet for persons living in community-based residences is a complex matter. Availability and accessibility of healthy food is crucial, but promoting a healthy diet also requires time and competence among caregivers and staff (26). The heterogeneous group of persons belonging to the ID category have been marginalized (27). However, there are changes taking place with increased awareness, and the Norwegian authorities have supported projects addressing nutritional issues for persons with ID the later years. The right to health, education and political influence has also been addressed in government documents (https://www.regjeringen.no/no/dokumenter/nou-2016-17/id2513222/). Several issues addressed in the Norwegian debate echo many of those addressed in the above cited UN documents.

In previous studies, we have addressed the issue of a human right-based approach in nursing homes for the elderly regarding nutritional issues (28). Many of the issues we have focused on for elderly persons living in community residencies are also relevant for those working with persons with ID. For persons with ID, there are, however, issues that demand special attention, such as staff competence on ID and specific syndromes, nutritional competence and tailored practical measures to improve nutrition. Still, in a human rights perspective, the issues of staff as duty bearers and the residents as right holders provide us with a set of tools to evaluate the quality of the services offered to this heterogeneous and vulnerable group of people.

## Issues that needs to be addressed

In summary, many persons with ID are obese or underweight. To improve their health, there is a need for improved national services and for more research on lifestyle and nutritional issues in persons with ID. From a human rights perspective, these issues must be put on the agenda both in relevant UN fora and in the respective nations’ health policies. Issues that must be addressed include the following:

1. The human right to adequate food for persons with ID needs to be more specifically addressed.
2. Improve national data regarding ID and health, including data regarding the number of persons with ID in the different countries and regions, and how many have ID caused by genetic diseases and syndromes. Further, register studies may provide unique data on special health risks and needs relevant for the various types of ID.
3. Improve the nutritional care in national services. To achieve this, there is a need of increased knowledge about healthy diet in staff working with persons with ID and to develop guidelines and manuals for how national services should work for implementing a healthy lifestyle and diet for ID.
4. Increase international research collaboration.
5. Develop nutritional-related outcomes measures that are adjusted for use in persons with ID.
6. Perform preventive nutritional studies, in order to promote longevity and long-term health.
